# Evaluation of the *Tsima* community mobilization intervention to improve engagement in HIV testing and care in South Africa: study protocol for a cluster randomized trial

**DOI:** 10.1186/s13012-016-0541-0

**Published:** 2017-01-17

**Authors:** Sheri A. Lippman, Audrey Pettifor, Dumisani Rebombo, Aimée Julien, Ryan G. Wagner, Mi-Suk Kang Dufour, Chodziwadziwa Whiteson Kabudula, Torsten B. Neilands, Rhian Twine, Ann Gottert, F. Xavier Gómez-Olivé, Stephen M. Tollman, Ian Sanne, Dean Peacock, Kathleen Kahn

**Affiliations:** 1 Center for AIDS Prevention Studies, Department of Medicine, University of California San Francisco, 550 16th Street, 3rd Floor, San Francisco, 94158-2549 CA USA; 2 MRC/Wits Rural Public Health and Health Transitions Research Unit (Agincourt), Faculty of Health Sciences, School of Public Health, University of the Witwatersrand, 27 St Andrews Road, Parktown 2193, Johannesburg, South Africa; 3 Gillings School of Global Public Health, University of North Carolina, 135 Dauer Dr., Chapel Hill, 27599 NC USA; 4 Sonke Gender Justice, 4th Floor Westminster House, 122 Longmarket Street, 8001, Cape Town, South Africa; 5 Department of Population Health, London School of Hygiene and Tropical Medicine, Keppel Street London WC1E 7HT, London, UK; 6 Clinical HIV Research Unit, Department of Medicine, Faculty of Health Sciences, University of the Witwatersrand, Helen Joseph Hospital, Perth Road, Westdene, 2092, Johannesburg, South Africa; 7 Umeå Centre for Global Health Research, Division of Epidemiology and Global Health, Department of Public Health and Clinical Medicine, Umeå University, 90187, Umeå, Sweden; 8 Division of Social and Behavioural Sciences, School of Public Health, University of Cape Town, Falmouth Rd, Observatory 7925, Cape Town, South Africa

**Keywords:** Cluster randomized trial, Community mobilization, South Africa, HIV testing, Engagement in care, Retention in care, Treatment as prevention

## Abstract

**Background:**

HIV transmission can be decreased substantially by reducing the burden of undiagnosed HIV infection and expanding early and consistent use of antiretroviral therapy (ART). Treatment as prevention (TasP) has been proposed as key to ending the HIV epidemic. To activate TasP in high prevalence countries, like South Africa, communities must be motivated to know their status, engage in care, and remain in care. Community mobilization (CM) has the potential to significantly increase uptake testing, linkage to and retention in care by addressing the primary social barriers to engagement with HIV care—including poor understanding of HIV care; fear and stigma associated with infection, clinic attendance and disclosure; lack of social support; and gender norms that deter men from accessing care.

**Methods/design:**

Using a cluster randomized trial design, we are implementing a 3-year-theory-based CM intervention and comparing gains in HIV testing, linkage, and retention in care among individuals residing in 8 intervention communities to that of individuals residing in 7 control communities. Eligible communities include 15 villages within a health and demographic surveillance site (HDSS) in rural Mpumalanga, South Africa, that were not exposed to previous CM efforts. CM activities conducted in the 8 intervention villages map onto six mobilization domains that comprise the key components for community mobilization around HIV prevention. To evaluate the intervention, we will link a clinic-based electronic clinical tracking system in all area clinics to the HDSS longitudinal census data, thus creating an open, population-based cohort with over 30,000 18–49-year-old residents. We will estimate the marginal effect of the intervention on individual outcomes using generalized estimating equations. In addition, we will evaluate CM processes by conducting baseline and endline surveys among a random sample of 1200 community residents at each time point to monitor intervention exposure and community level change using validated measures of CM.

**Discussion:**

Given the known importance of community social factors with regard to uptake of testing and HIV care, and the lack of rigorously evaluated community-level interventions effective in improving testing uptake, linkage and retention, the proposed study will yield much needed data to understand the potential of CM to improve the prevention and care cascade. Further, our work in developing a CM framework and domain measures will permit validation of a CM conceptual framework and process, which should prove valuable for community programming in Africa.

**Trial Registration:**

NCT02197793 Registered July 21, 2014.

## Background

In 2014, UNAIDS established targets in hopes of reaching the end of the HIV epidemic by 2030. The targets dictate that by 2020, 90% of all people living with HIV should know their HIV status, 90% of all people with diagnosed HIV infection will receive sustained antiretroviral therapy (ART), and 90% of all people receiving ART will achieve viral suppression [1]. This 90-90-90 framework was largely the result of ground breaking research demonstrating that HIV transmission can be decreased substantially by reducing the burden of undiagnosed HIV infection and expanding early and consistent use of ART. [2, 3] Treatment as prevention (TasP), or treating an HIV-positive person with ART to reduce their HIV viral load and thus reduce the risk of forward transmission of the virus to a negative partner, is the key to ending the HIV epidemic [4, 5]. In high HIV prevalence countries like South Africa (adult national HIV prevalence estimated at 16.9%) [6, 7], observational studies have demonstrated that residents of communities with greater ART coverage have a lower risk of HIV acquisition [8].

To activate TasP in high prevalence settings and reach 90-90-90 targets, communities must have access to HIV testing and treatment and also be motivated to know their status, engage in care if they test HIV positive, and remain in care, including medication adherence to ensure viral suppression. Currently uptake of testing, linkage to care, and treatment falls short of that needed to significantly decrease new infections, despite widespread availability of HIV testing and care services. National data from South Africa indicated that in 2012 while 65.0% of the population reported ever testing for HIV, with approximately 40% having tested in the last year, only 37.8% of HIV-positive men and 55.0% of HIV-positive women knew their HIV status [9]. Though South Africa has the largest ART program in the world, only an estimated 52% of eligible patients are on treatment [10], and only an estimated 31% of those living with HIV have initiated treatment [7]. Furthermore, population-based data from rural areas in the North West province in 2014 indicate that less than half of those on ART are virally suppressed [11]. At current rates of testing and treatment coverage, South Africa will fall far short of meeting the 90-90-90 goals.

Primary barriers to testing uptake and engaging in HIV care are related to social and structural factors that shape clinical care seeking—these include HIV-related stigma and fear of disclosure, fears around treatment side effects, male gender norms that discourage men from engaging in care, and a general lack of community awareness about the benefits of HIV care and treatment [12]. HIV-related stigma leads to delays in HIV testing and treatment as well as poor adherence to clinic visits and medication [13–16]. Similarly, fear of ART side effects and misconceptions around the benefits of early diagnosis, care and treatment can keep people who are living with HIV/AIDS (PLWHA) out of care [17, 18]. Finally, gender norms are a major social barrier for testing, linkage to care and treatment for men, as traditional gendered conceptualizations dictate that it is unmasculine to seek care, resulting in men accessing health care services at lower rates than women [19–22]. Men also access ART at a far lower rate and at more advanced stages of disease compared to women [23–27].

Tackling social barriers to HIV prevention, testing and linkage to care, requires building a sustained, community-wide response [28]. Community mobilization (CM) has significant potential to improve testing uptake and linkage to and retention in care by addressing the social barriers to engagement with HIV care [12, 29, 30]. There is strong evidence that CM can improve behavioral outcomes, such as consistent condom use, by addressing discrimination, creating social cohesion, and extending social participation and networks for target communities [28, 31–38]. CM has successfully changed inequitable gender norms through engaging men to question traditional masculinity and support each other to change social inequalities [37, 39–43]. In the large Project Accept trial, CM markedly improved HIV testing uptake by changing community norms around HIV testing through enhanced community participation, raising community awareness, and partnership building [44, 45]. UNAIDS has designated CM as a critical enabler—or an activity that is necessary to support the effectiveness of programs. [46] Despite this, CM strategies for linkage to and retention in HIV care have not been rigorously defined or evaluated, with few conceptual frameworks that provide a theoretical basis for program design. In fact, few interventions have improved linkage to and retention in HIV care in resource poor settings, despite the urgent need [47–49].

To address this gap in interventions to improve linkage to and retention in HIV care on a community level, we designed a cluster randomized trial to implement and evaluate a theory-based CM intervention conducted and allocated at the community level that addresses known social barriers to engagement in HIV care. We aim to determine whether the intervention decreases undiagnosed infections and improves linkage to and retention in HIV care among adults aged 18–49 years in the intervention communities as compared to adults 18–49 in the control communities, with the overall goal of reducing new HIV infections and improving health outcomes of those living with HIV. We also aim to understand the mechanisms of action of this mobilizing program through our community surveys and determine what comprises the ideal format for delivery of the program. This manuscript describes the trial protocol.

## Methods/design

### Study site and population

This study is being conducted in the Agincourt sub-district of Bushbuckridge in rural Mpumalanga province of South Africa—the area is covered by a health and socio-demographic surveillance system (Agincourt HDSS) established in 1992 and run by the Medical Research Council/Wits University Rural Public Health and Health Transitions Research Unit (Agincourt). Through the annual household and vital events update, the unit maintains a detailed database and sampling frame, including geo-coding, of over 20,000 households and over 115,000 resident individuals living in 31 fully enumerated villages, 15 of which are participating in this study [50]. The current study focuses on HDSS residents aged 18–49 years.

**Fig. 1:**
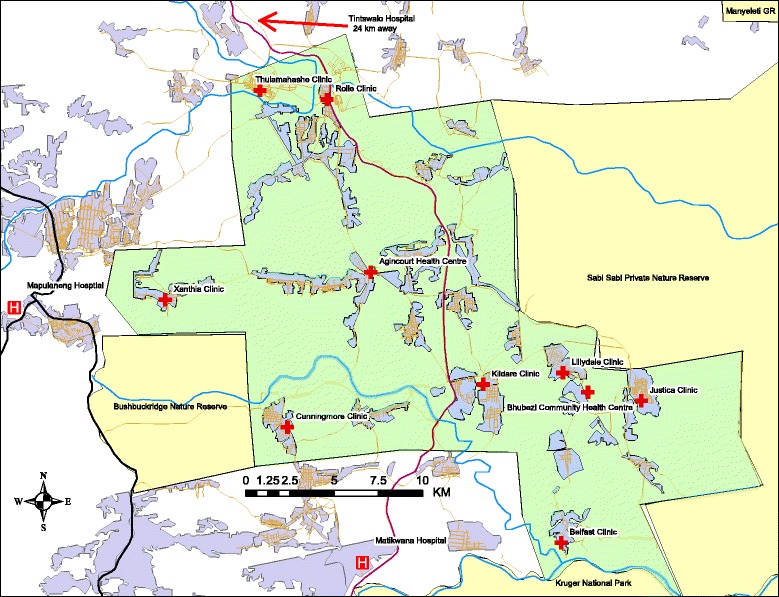
Map of the Agincourt Health and Socio-Demographic Surveillance System (Agincourt HDSS) and surrounding area

Population-based HIV prevalence among the adult population 15 years and above in the Agincourt HDSS was 19.4% in 2010–2011 and peaked at over 45% among 35–39-year olds [51]. HIV testing and clinical care for HDSS residents is provided by nine public health facilities and one public-private-partnership community health center located within the study area. These ten facilities serve multiple communities throughout the HDSS. As community members commonly receive services outside their village, the study includes monitoring HIV services provision at all ten health facilities (Fig. 1).

### Specific aims

The CM intervention is being evaluated by comparing uptake of testing, linkage, and retention in care in 15 communities randomized to either an intervention condition (*n* = 8) or a control condition (*n* = 7) located within the Agincourt HDSS. To measure outcomes in each village, we are linking the longitudinal population-based HDSS data to a health facility-based electronic clinical tracking system used in all HDSS health facilities. The linkage of the health facility and census data allows us to establish an open cohort of approximately 34,000 18–49-year-old residents that includes exposure data (village of residence) as well as outcome data (HIV-related clinical care). Using these data, we will evaluate the effects of the CM intervention by village randomization arm as well as characterize the HIV care cascade in a high prevalence population. We will also evaluate the conceptual model, including the hypothesized CM processes [52], through community surveys to monitor intervention exposure and community level change using validated CM measures [53]. The specific aims of the study include:

#### Aim 1, testing

To determine whether uptake of HIV testing among residents of communities receiving a community mobilizing intervention is higher than residents of control communities.

#### Aim 2, linkage

To determine whether linkage to care is higher among residents of intervention versus control communities as measured by (a) proportion of those testing HIV positive who complete CD4 staging within 3 months of diagnosis and (b) the proportion of those eligible for treatment who initiate ART within 3 months of CD4 staging.

#### Aim 3, retention

To determine whether retention in care is higher among people in intervention compared to control communities, including (a) proportion of ART initiated patients not defaulting from care (with no more than a 90-day gap in treatment during 12 months) and b) those ineligible for ART returning for CD4 staging at 6-month intervals.

#### Aim 4, mechanisms

To explore changes in community mobilization domains as well as how differences in each domain associate with changes in study outcomes (testing, linkage, and retention) over time.

### Community randomization

Communities involved previously in community mobilizing activities (*n* = 12), those not yet fully enumerated in 2014 (*n* = 3), and with fewer than 500 permanent residents (*n* = 1) were not eligible to participate, leaving 15 communities to randomize. In order to achieve a balance among villages on the key covariates at baseline, a restricted or balanced randomization approach [54] was used, taking into account population size, distance to closest health facility, average levels of SES based on household assets [55], temporary migration, female-headed households, and baseline community mobilization scores [53], collected as part of a survey in 2014. Cluster allocation cannot be concealed as CM events and activities are held in public spaces. Further, village leaders were consulted about the trial, consented for their villages to participate, and were present for the allocation. A randomization event was carried out with community representatives. A biostatistician unfamiliar with the area and the villages generated 50 different grouped combinations of intervention and control villages, and these combinations were printed on strips. The assignment was revealed by asking a community volunteer to pick the winning randomization scheme out of a bucket containing all 50 possibilities. The program was then launched in the presence of local leaders and community members.

### Intervention design

**Fig. 2:**
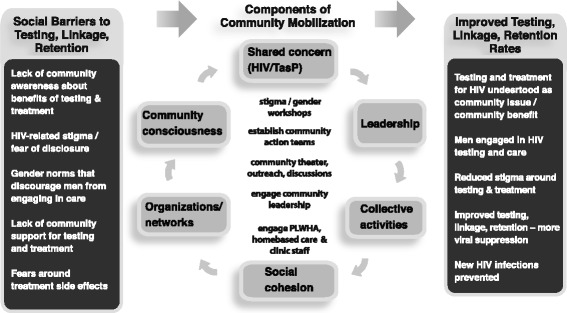
Conceptual framework of the intervention, community mobilization components, and study goals

Overall, our mobilization model adheres to the theory that social barriers necessitate social change solutions—those which move beyond merely providing services or empowering individuals to use services and that instead construct a collective response out of a group of individuals [28]. As a result, our intervention addresses social barriers to testing, and linkage to and retention in HIV care, specifically, poor awareness or understanding of HIV care; fear and stigma associated with HIV infection, clinic attendance, and disclosure; lack of social support; and gender norms, particularly those that deter men from accessing care. These content areas are addressed by implementing activities across the intervention villages that engage communities in social change—specifically activities that map onto six CM domains distilled from the social sciences literature and validated in the study area (see Fig. 2) [52]. The six domains of mobilization that are being addressed for CM to successfully impact HIV prevention are (1) a shared issue or concern that is the target of change, (2) community sensitization or building of critical consciousness, (3) an organizational structure with links to groups/networks, (4) leadership (individual and/or institutional), (5) collective activities/actions, and (6) community cohesion. These domains represent community factors integral to social change that must be addressed or modified for mobilization to occur and to impact behaviors, social norms, and health outcomes in the communities.

The program is being carried out in partnership with Sonke Gender Justice, a South African non-governmental organization (NGO) dedicated to activism and health programming at the intersection of HIV and gender equity. Intervention activities and workshop content have been adapted from material that has been compiled, pilot tested, and successfully utilized in our previous collaborative work on community mobilization to address gender norms [56]. In preparation for the intervention we both refined previously designed activities to fit the new programmatic content and further developed new activities addressing social barriers to testing, linkage to, and retention in care. New activities, workshop content, print materials, and short films were developed to specifically address the issue of treatment as prevention (TasP) and HIV treatment literacy. We also added more in depth content on stigma and social norms surrounding HIV and health care utilization. Over 16 community-based activities have been developed, standardized and manualized into a toolkit of community mobilizing activities; 50 modules covering 7 themes have been included in a workshop manual; and a handbook for creation of community mobilizing teams has been developed and refined [57]. The mobilizing team named the intervention “Tsima ra rihanyu,” which means “working together to plow the fields for health” in the local language.

Tsima ra rihanyu is being implemented over 3 years by a team of 16 community mobilizers with two intervention coordinators, a logistician, and a member of the senior staff at Sonke Gender Justice. Mobilizers were selected from intervention communities and assigned to work in their own villages whenever possible. These individuals underwent extensive training from master trainers at Sonke on all intervention activities and are supervised by the intervention coordinators. As part of the Tsima ra rihanyu activities, the mobilizers also identified and trained community action teams (CATs) in each village. CATs are volunteers from the community who engage with the CM activities and lead mobilization efforts in their community; mobilizers work closely with CATs as they implement activities. This CAT model has been successfully utilized by Sonke in previous efforts.

Intervention activities aim to open dialogue and community discussion about testing, linkage to and retention in care through CM activities, harnessing networks for message dissemination, and forging organized community action through establishing CATs, engaging leadership, and fostering community cohesion to support people living with HIV (see Table 1). The CM team, along with the CATs, are carrying out activities to generate *a shared concern and raise consciousness* around engagement in HIV prevention and care, with a focus on the importance of testing, linkage to and retention in care for individuals, and overall community health. Activities to *raise consciousness* include intensive small group workshops, community outreach, including theater, debates and discussions, and digital story workshops—an event where real stories of individuals’ experiences with testing and engagement in care are told and then screened to generate discussion, and engagement with clinic staff. We launched the intervention with smaller group activities and workshops that focus on raising community consciousness and generating a shared concern around testing, linkage to care and treatment, and identifying CAT membership in order to set the stage for implementation of the full complement of CM activities. CATs act as community/clinic liaisons and work with the CM team to *engage local leadership* and stakeholders in these discussions and also work with existing *organizations and networks*, such as support groups or home-based care groups, as part of efforts to build community consciousness and shared concerns around TasP. As a next step, the CATs and mobilizers work to *lead collective actions* around testing, linkage, and retention such as designing community murals, hosting soccer tournaments, and other appropriate community events. In these events, efforts to build social cohesion and engage leadership are emphasized. Illustrative activities are included in Table 1.

**Table 1:** Intervention activities addressing social barriers to treatment as prevention, mapped onto community mobilization domains

	Social barriers to TasP			
CM domains	Stigma related to testing, care, and treatment	Increased knowledge (diminish fears) about testing and treatment	Gender norms around testing and treatment	Social support around testing and treatment
Building shared concerns and community consciousness	*2-Day intensive and single session workshops -focus on community stigma around testing/treatment *Door to door outreach *Digital stories and film screening *Engaging with clinic staff	*Street theater-focused on barriers to testing/treatment *Digital stories and films on testing and care experiences *Door to door outreach *Educational events	*2-Day and intensive and single session workshops on gender norms and the benefits/barriers to men engaging in testing and care *Street theater addressing gender norms	*2-Day intensive and single session group workshops-focused on community barriers to testing and treatment *Door to door outreach *Digital stories screening
Engaging leadership and stakeholders (includes traditional leaders, religious leaders, clinic leaders, other community stakeholders)	*Engaging leaders around importance of TasP and barriers in community *Intensive workshops with leaders on HIV stigma, testing/treatment *Pursuing leadership commitments to achieving village testing and treatment targets (goal setting)	*Engaging leaders around importance of TasP and barriers in community *Monthly 1-day small group workshops focused on benefits of testing and treatment in community	*Engaging leaders around the importance of men engaging in health care *Seek support from leadership to enter places of work, taverns, places where men congregate	*Identifying home based care support groups in CAT development *Engaging leaders in creating support networks for testing and treatment as a community benefit *Goal setting with leadership
Orgs/networks (includes NGOs, CBOs, CATs, other family or community groups/networks)	*Working with the key groups to openly support and include testing and treatment in their work in the communities	*Working with key groups to understand how testing and care can improve community well-being *Message dissemination through networks.	*Working with the key groups (employers, small businesses, sport teams, etc..) to support engagement of men in testing/care *Dialogues with church leaders addressing support for male testing/care	*Identifying home-based care groups and PLWH in CAT development *Partnering with support and treatment networks *Founding community support groups
Collective action	*Murals that address stigma related to testing and treatment *Events to support testing campaigns/treatment access *Encouraging Tsima members and affiliates to HIV test together	*Soccer tournaments that highlight importance of testing and treatment *Events to support testing campaigns/treatment access *Home education (open house) events for networks/groups	*Community events/forums that address gender norms and accessing HIV care *Murals that address gender norms and HIV care	*Community events conducted by CATS, PLWH, and home-based care groups to increase community support around TaSP *Encouraging Tsima members and affiliates to HIV test together
Social cohesion	*Visible community support (events/forums) to reduce stigma–working with PLWH *Community events to dialogue around how stigma affects the community and how communities can respond	*Identifying home based care groups and PLWH in CAT development–providing safe space for discussion and support *Events to support stronger care and treatment networks	*Dialogues with men’s groups or associations–addressing male support for testing/care. *Building CATs with men, including men LWH	*Work with home-based care and CATS to establish PLWH support networks *Extend networks and activities to families *Join CATs, local leaders, and clinic staff in testing and treatment strategies.

### Intervention monitoring

Intervention activities are continuously monitored in order to quantify intervention consistency (fidelity) as well as dosage and reach by village. Data collection forms are completed by mobilizers and CAT members in order to capture which activities are undertaken in the communities and how many residents were in attendance. Monitoring reports are generated monthly and quarterly to assess the estimated proportion of residents exposed to the CM intervention in each village and to ensure the team is reaching intervention coverage targets. With this system capturing data in real time, any indication that a village may fall below a given threshold (i.e., reach 25% of the village population with workshops annually) can be noted early and corrective action taken to ensure the village receives additional support from the mobilizing team. In our past experience implementing CM, this system has allowed us to ensure that all activity targets are met within the specified timelines.

### Data collection

**Fig. 3:**
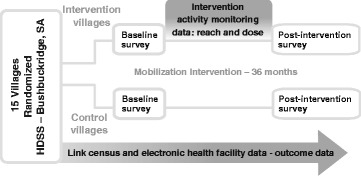
Study design and data collection components

Continuous data collection of HIV-testing and care delivery visits is underway at all ten health facilities in the study area by a team of trained data capturers stationed at the facilities, who both obtain written informed consent from patients to capture clinical visit data and conduct the HDSS-health facility data linkage in real time (Fig. 3). The data linkage is done using deterministic and probabilistic approaches. After obtaining written informed consent from a new patient, the data entry clerk collects a set of identifiers that are used to search the Agincourt HDSS data. Linkage is attempted using national ID number and, if a match is not found, then a second attempt is performed using a combination of mobile phone number, first name, and date of birth. If a match is still not found, a search is done using an algorithm based on the Fellegi-Sunter probabilistic record linkage model [58]. The key identifiers include name, surname, age or date of birth, sex, village of residence, and name of another person living in the household [59]. When potential matches are found, they are reviewed in the presence of the patient to confirm his or her identity. In addition to identification of the patient in the population database, the data entry clerk extracts clinical information from the patient’s file and logs all follow-up visits, entering clinical information directly into a laptop hosting the linkage system. The linkage system encrypts all data as soon as it is entered; identifying information (e.g., name and phone numbers) are excluded from analytic datasets.

To measure coverage of the intervention and changes in the CM domains and other key variables in each community over time, a cross-sectional, population-based survey was administered prior to the intervention and a second survey will take place following the intervention (Fig. 3). The surveys aim to capture 1200 randomly sampled adults, aged 18–49 years, from the HDSS population with approximately 75 people sampled in each of the 15 study villages at both time points. Eligibility criteria for participation in community surveys include residence in the home (spending a majority of nights at home), being 18–49 years of age, and having lived in the study area for at least 9 of the past 12 months. Surveys are interviewer-administered in the participant’s home using computer assisted personal interview (CAPI). Only one individual per household can be selected. Fieldworkers visit the selected homes to confirm eligibility and explain the study. Written informed consent is obtained from all participants prior to the survey; both consent and the survey are offered in the local language, Shangaan, or English. All procedures were approved by the Institutional Review Boards at the University of North Carolina-Chapel Hill and the University of California-San Francisco, the Human Research Ethics Committee (Medical) at the University of the Witwatersrand in South Africa, and the Mpumalanga Department of Health and Social Development Research Committee.

### Study measures

#### Primary exposure

Residence in an intervention or control community is the primary predictor variable for all analyses. Community of residence will be determined using the HDSS data. Household and vital events data are updated annually and include births, in- and out-migrations and deaths. Although few individuals are expected to move within the study area over the course of the study, for those who do move, exposure will be determined dynamically over the course of the study such that individuals will be considered exposed if they were resident in an intervention community in the time period immediately prior to the outcome. Table 2 summarizes the quantitative data elements that include exposures, outcomes, mediators, and social and demographic co-variates.

**Table 2:** Summary of quantitative data elements

Domain	Instrument/measure	Data source and frequency
Primary exposure		
Village	Village of residence (binary; intervention vs. control village)	HDSS-annual
Primary outcomes		
Testing uptake (aim 1)	Binary: tested/untested past 12 months, among HIV-negative or unknown status residents	Electronic health facility records-ongoing
	Binary: known HIV status–either confirmed positive or tested within the last 12 months	Electronic health facility records-ongoing
Linkage to HIV care (aim 2)	Binary: received baseline CD4 results and evidence of follow-up care (additional CD4, viral load, or treatment initiation) within 3 months of testing HIV positive	Electronic health facility records-ongoing
	Binary: treatment initiation within 3 months of positive diagnosis among those eligible for ART	Electronic health facility records-ongoing
Retention in HIV care (aim 3)	Binary: HIV patients on or initiating ART who have no more than a 90-day gap in medication received in the 12-month period (no defaulting).	Electronic health facility records-ongoing
	Binary: HIV patients not ART eligible^a^ who have a repeat CD4 test 6–12 months after initial CD4	Electronic health facility records-ongoing
Meditator/mechanism		
Community mobilization	Six domains of community mobilization measure [53]	Population-based surveys (years 1 and 5)
Covariates		
Demographics	Age, SES, gender, migration status	HDSS-annual
Social norms	Stigma [81]; gender norms [41]; community support for HIV testing and treatment [53]	Population-based surveys (years 1 and 5)
Secondary outcomes		
Testing (aim 1)	Median CD4 of people initiating ART (to explore earlier testing, entry into care)	Electronic health facility records-ongoing
Re-engagement in care (aims 2 and 3)	Patients out of care (not retained) who are re-engaged in care (have a CD4 test/initiate or re-initiate treatment).	Electronic health facility records-ongoing
Viral suppression	Proportion of residents with viral load <400 copies/ml	Electronic health facility records-ongoing
Secondary exposure		
Intervention coverage	Reported exposure to intervention events	Population-based surveys (years 1 and 5)

^a^Prior to September 2016, patients were considered ART eligible with one or more of the following criteria: pregnancy, CD4 count lower than 500 cells/mm [3], active tuberculosis, WHO stage 3 or 4, or initiation of ART per clinician discretion. Universal treatment was planned to be instituted in September 2016; therefore, all HIV-positive individuals will be considered treatment eligible after this date; the pre-ART definition will only apply to the period prior to implementation of the universal treatment guidelines

#### Primary outcomes

For each aim, the primary source of data for outcomes is the electronic health facility data linked to the HDSS. An electronic record will be created for every resident of the 15 study villages captured in the population database; with this approach, we will treat the entire eligible (18–49-year old) population in the area as an open cohort, assuming no clinic visits were made if none were captured for a given individual. Study outcomes include HIV testing uptake in the past 12 months among residents of negative or unknown status (aim 1); linkage to HIV care, or having returned to the clinic following diagnosis for ART initiation or follow-up care (aim 2); and retention in care, defined as remaining on ART with no more than a 90-day gap in coverage over a 12-month period (aim 3).

#### Mediators: community mobilization

Quantitative measures of CM will be collected in the two community surveys; CM is comprised of six hypothesized domains, outlined in Table 1. Questions regarding a *shared concern* about HIV/AIDS are designed to capture whether members of the community define HIV testing and care as important, problematic, and mutable issues, and whether they believe they can work together to address HIV. The scale for *critical consciousness* is designed to capture whether members of the community are undergoing processes of critical reflection and dialogue about their circumstances and ways to improve their lot. Questions about *leadership* capture leadership capacity, diversity, responsiveness, accessibility, and support of collective decision making. Questions regarding *organizations and networks* are designed to capture the existence and influence of community-based organizations, groups, and networks that can serve as a resource in mobilizing—both for exchange and diffusion of ideas and as a structure that can be utilized for community organizing. Questions regarding *collective action* are designed to capture the presence, breadth, and quantity of collective activities in the villages aimed at social change. Finally, questions about *social cohesion* capture the level of working trust in a community. Scales performed extremely well for all six CM domains in our 2012 survey [53].

### Analysis

One-way frequency tables for all variables and measures of central tendency and variability for continuous variables will be generated to perform range checks, quantify the amount of missing data, and yield valuable descriptive findings that will further characterize the care cascade in the study population. These analyses will also be stratified by randomization group (i.e., intervention versus control) using cluster-based two-group comparison methods (e.g., Rao-Scott chi-square) to check the equality of intervention and control group covariates at baseline. If the intervention and control groups are found to differ significantly at baseline on one or more covariates (e.g., gender; mean age of village residents), we will use methods based on the Rubin causal model (e.g., propensity scores, targeted maximum likelihood estimation, double-robust estimation) [60] to obtain the desired marginal effect estimates under the counterfactual assumption of balanced groups [61–65]. We will assess and document the amount of missing data from health facility systems and community surveys. In the unlikely event that a variable has >5% of missing data, we will address incomplete data via either inverse probability of censoring weights (IPCW) or use multiple imputation (MI) [66], as it makes the relatively mild assumption that incomplete data arise from a conditionally random (MAR) mechanism rather than the completely random process (MCAR) assumed by ad hoc methods such as list-wise data deletion [67]. For MI, auxiliary variables will inform multiple imputations to increase the likelihood of meeting the MAR assumption [68, 69].

We hypothesize that following the intervention, for the intervention community relative to the control community residents:

1. The odds of HIV testing will be higher (specific aim 1);
2. The odds of linkage to care will be higher (among those HIV-infected; specific aim 2);
3. The odds of retention in care will be higher (among those HIV-infected; specific aim 3); and
4. The community means of the six domains of community mobilization will be higher and associated with individual level outcomes (specific aim 4).

Primary analyses will follow an intent-to-treat (ITT) approach and will be unadjusted except for intervention group, and, for longitudinal analyses, time, and the group-by-time interaction. Individual will be the unit of analysis. Our principal interest is to estimate the marginal or population-average effect of the intervention on each outcome rather than the effect for a hypothetical average subject [70]. Moreover, within-subject correlations among outcomes are considered nuisance parameters rather than quantities of interest to be modeled explicitly. Accordingly, generalized estimating equations (GEE) will be used to perform the proposed primary longitudinal analyses, including planned time-averaged comparisons of post-baseline measurements across the intervention and control arms to test primary hypotheses 1–3 listed above. Alpha will be set at .05 for these planned comparisons of each separate outcome. Any additional post-hoc comparisons (e.g., paired comparisons of the control and intervention arms at each time point) will maintain a nominal alpha level of .05 through the use of simulation-based stepdown multiple comparison methods [71]. GEE accounts for correlation of repeated measurements from the same research participant via computation of a working correlation matrix and yields the desired marginal population-average effect estimates [72]. Village ID will be included as a nested fixed effect or via alternating logistic regression (ALR) to account for clustering of persons nested within villages. GEE estimates are consistent even if the correlation structure is mis-specified, though GEE’s statistical efficiency improves as the working correlation structure more closely approximates the actual correlation structure [73]. Thus, several suitable working correlation structures will be considered (e.g., unstructured, AR(1), exchangeable) [74]. The QIC or a similar statistic will be used to select the final working correlation structure [75]. Robust Huber-White “sandwich” standard errors will be used to obtain correct inferences even if the chosen correlation structure remains slightly mis-specified.

For hypothesis 4, cross-sectional population data will be collected through representative community surveys. Summary measures of the CM domains before and after the intervention for each village will be calculated from this population representative sample. GEE will be used to model the effects of the pre-post differences in village-level CM domains on individual residents’ HIV testing, and, for HIV-positive individuals, linkage and retention to care using the same overall modeling approach as outlined above. To examine the relative importance of each of the six CM domains, initial models will include the pre-post change scores for each of the six domains in a single model. Subsequent analyses will isolate the marginal effects of each domain via G-computation or other substitution-based estimation strategy [60, 76].

Secondary analyses will extend GEE models to incorporate additional covariates of interest drawn from the HDSS data (e.g., age; gender) and examine their interactions with intervention assignment to ascertain statistical moderation. Secondary analyses will also evaluate whether the pre-intervention/post-intervention village-level difference in the six community mobilization measures mediate the relationships between intervention village residence and the primary outcomes. Statistical mediation will be assessed using the causal inference-based approach of Valeri and VanderWeele, which yields optimal estimates of indirect effects in the presence of binary outcomes and moderator-mediator interactions [77]. M*plus* will be used to implement these mediation analyses because it allows for causal mediation assessment incorporating robust standard errors to address clustering of individuals within villages [78]. Significant indirect and total effects of the intervention on testing, linkage, and retention accompanied by non-significant direct effects will signify mediation in this context.

### Power

Minimum detectable differences were calculated according to the methods for proportions in cluster randomized trials proposed by Hayes and Moulton [54], yielding conservative effect size estimates based on clustering by villages. Based on the HDSS data, there are approximately 30,000 adults 18–49 in the study area. We assumed an HIV prevalence of 33% in the adult target population (18–49-year old) [51]. If we conservatively estimate that testing is constant at 35% of the population, at least a third of those tested each year were not tested previously in the study period, and that test positivity is 15% (again a conservative estimate), 449 cases of HIV would be newly identified each year. We further assumed that approximately 50% of individuals who present for CD4 staging would be eligible for HIV treatment under 2014 guidelines. Approximately 6000 individuals were known to be on treatment at clinics in this area in 2014. Our survey data from 2012 suggests that HIV testing in this region exhibits an intra-class correlation of 0.014. In adjustments of power calculations for clustering of outcomes by village, we conservatively assumed an intra-class correlation of 0.05. Table 3 includes outcome estimates for the control proportions based on current literature [18, 26, 27, 68, 79], and the target (intervention) proportions with the minimum increase in proportions that would be detectable as a statistically significant change at alpha of 0.05 and power of 0.80, given the above assumptions. In 2016 (1 year into data collection), the South African government announced universal HIV treatment. This should increase our sample size to assess initiating ART (aim 2) and retention in care (aim 3) in the second and third years of the study.

**Table 3:** Power calculations–minimum detectable effects

Outcomes: proportion of population	Current estimate	Intervention group target	Minimum detectable difference (proportion)
Testing; tested in past 12 months	35%	60%	19%
Linkage; undergoing CD4 staging within 3 months of positive test	65%	85%	18%
Linkage; eligible for ART who initiate treatment within 3 months	60%	80%	19%
Retention; HIV positive who remain in care at 12 months	50%	70%	19%

## Discussion

The Tsima ra rihanyu program is built on a theory-based CM model that aims to remove the major social barriers to successful participation in the HIV care cascade and ultimately activate the promise of TasP. The cluster-randomized research design and the linked data sources including a measure of CM administered through community surveys will provide a robust structure within which to evaluate the intervention and simultaneously, provide insight as to the mechanisms of action in improving testing, linkage, and retention. In addition, because the study is embedded in an HDSS, we will have true denominators for our estimates and will therefore generate much needed population-based data contributing to improved characterization of the HIV care cascade. These data will also provide an opportunity to optimize program design through targeting groups with the greatest need of care support.

This study focuses on community approaches to improving uptake of HIV testing and care and is not focused on directly mitigating health facility-level barriers to care, including quality of HIV care, drug stock-outs, long wait times, or lack of confidentiality. However, the intervention design does not prohibit mobilization teams and/or CATs to plan activities at the clinics or with the clinics and does encourage partnerships with clinic leadership as primary stakeholders in improving the care cascade. Because intervention and control populations will be accessing the same group of clinics and because varied quality of care across the health facilities and different levels of involvement in the intervention by health facility could affect the study outcomes, the team agreed that any intervention activities that target health facility leadership or address health facility services will be conducted in all study health facilities, in order to ensure that potential effects of clinic-based activities will be consistent across all villages. Additionally, to address basic health facility functionality, and mitigate any health facility-specific influence on study outcomes, the team has partnered with the local PEPFAR partner, Right to Care, to work with all study area health facilities to ensure that best practices for HIV prevention, testing, care, and treatment are in place and that monitoring systems and data capture are standardized and that supplies, including test kits and drugs, are available.

The Tsima ra rihanyu program is also rooted in community collaboration and feedback, with community participation in setting goals as well as monitoring progress towards reaching those goals. A key strength of this intervention is the ability to assess the care cascade in each community over time. In the first year of the intervention, we held goal-setting workshops with each intervention community to establish commitment and partnership towards improving HIV outcomes in the community. The concept of the UNAIDS 90-90-90 goals was introduced during these meetings and community leaders, and participants were able to both assess the current state of HIV testing and care outcomes in their community and establish annual community goals. Every 6 months, the team will hold a similar community meeting to monitor achievements towards reaching the goals and renew commitment and partnerships. Clinics are also receiving feedback on a regular basis, with a team discussion regarding where attrition is occurring in order for clinics to focus their efforts in improving clinical outcomes for their clients.

The study does have some limitations. The Tsima ra rihanyu program excludes use of mass media or large-scale public advocacy in order to minimize contamination in the context of an RCT. This omission will likely detract from the reach and impact of the intervention. Additionally, given substantial migration in the area, it is likely that some residents will be classified as lost to follow-up or out of care when they may have moved out of the area and remained in care. While information regarding changes in residency status (migration) and deaths are updated annually in the census and will be noted in the data, some residents captured in the electronic health facility data system may not present for return visits with no recorded reason for loss to follow-up. To remedy this, among those classified as lost, we will select a random sample of 10% to contact in order to determine their outcome, including whether they are seeking care elsewhere. This data will allow us to perform sensitivity analyses [80]. Additionally, we do not have current data on HIV status of all individuals in the community; therefore, the true number of residents living with HIV is unknown. We can, however, estimate the number based on previous population-based surveys in the area.

## Conclusions

This will be one of the first studies to measure the impact of community mobilization on uptake of testing, linkage, and retention in care and to identify the mechanisms through which CM impacts engagement in care. Results can inform community-based initiatives in areas of high prevalence where uptake of TasP is most critical.

## Trial status

The first cross-sectional survey was conducted between August-November, 2014. Data collection at health facilities in the study catchment area was rolled out across nine clinics between June and August 2015 and in an additional clinic from July 2016; clinic data capture is scheduled to continue in all clinics through 2018. Intervention activities commenced on 1st August 2015; the intervention is currently entering the second year of a 3-year intervention, scheduled to close out in July 2018.
